# Understanding the conservation patterns and molecular phylogenetics of human death receptors family through computational biology

**DOI:** 10.1007/s13205-013-0141-5

**Published:** 2013-05-25

**Authors:** Jinny Tomar, Chiranjib Chakraborty, C. George Priya Doss, V. K. Gera

**Affiliations:** 1 Department of Bio-informatics, School of Computer and Information Sciences, Galgotias University, Greater Noida, India; 2 Biotechnology Department, IILM Academy of Higher Learning, Knowledge Park, Greater Noida, UP India; 3 Medical Biotechnology Division, School of Biosciences and Technology, VIT University, Vellore, 632014 Tamil Nadu India

**Keywords:** Multiple sequences alignment, Molecular phylogenetics, Conservation patterns, Sequence logos, Computational biology

## Abstract

Human death receptors (TNFR1, FAS, DR3, DR4, DR5, DR6 and TNFBR), primarily from tumor necrosis receptor super family, play an essential role in the process of the extrinsic pathway of apoptosis. We performed conserved domain, amino acid residues analysis in which cysteine residues were found to be highly conserved for all the family members. Sixteen (16) highly conserved residues were observed in TNFR1, DR3 and TNFBR; and in case of Fas, only seven (7) residues are highly conserved. From molecular phylogenetics, we found that DR5 and DR4, TNFR1 and DR3 and TNFR1 and DR3 had the same point of origin. Alternatively, Fas was found to be distant from the rest of the death receptors. A network map was developed to explain these proteins are not only interlinked among themselves, but also interlinked with several other proteins. We have also observed from this system that scores of all the nodes ranges from 0.996 to 0.999.

## Introduction

The families of death receptor members belong to the tumor necrosis factor (TNF) or nerve growth factor receptor super family. Death receptors are known to initiate the process of the extrinsic pathway of apoptosis, and ligand-bound death receptors triggered the extrinsic pathway (Thorburn et al. 2004; Fesik 2000; Krueger et al. 2001) This death receptors family contains a sequence of 2–5 cysteine-rich extracellular repeats. These receptors also include an intracellular death domain (DD). This DD is required for transduction of the apoptotic signal. TNFR1, Fas, DR3, DR4, DR5, DR6, TNFBR (tumor necrosis factor beta receptor) are among the well-known human death receptors (Schulze-Osthoff et al. 1998; Thorburn et al. 2004; Krueger et al. 2001). The death receptors identify their ligand, based on structural uniqueness, which forms DISC (Death Inducing Signaling Complex) **(**Harper et al. 2003). Usually, functionality or transmission of the death signal is by the result of binding of the specific ligand to the death receptor which is then followed by the attachment of adaptor protein molecules. This results in activation of pro-caspases to mediate various signaling pathways, depending on the chosen adaptor protein (Sandra et al. 2005). These receptors have other non-apoptotic functions as well, for example, inflammatory responses, cell proliferation, cell immune responses, receptor internalization (Los et al. 2001; Algeciras-Schimnich et al. 2002). FasL binds to CD95 receptor, while TNF alpha and lymphotoxin forms the ligand receptor complex with TNFR1. TRAIL2 (Apo2L) receptor 1 and 2 forms close association with both the death receptors—DR4 and DR5, whereas DR3 only show its specificity by binding to Apo3L (Hongxia et al. 2009).

Evolutionary history can be studied through molecular phylogenetics and it can be explored further by molecular approach through amino acid sequencing in human (Kumar and Hedges 1998). It is a well recognized method for conservation genetics. This method plays a remarkable role in understanding the applied evolution; because genetic patterns can lead to an evolutionary process (Latta 2008; Chakraborty et al. 2012). Conservation especially evolutionary conservation of a protein sequence is directly linked with the conserved regions of protein sequence particularly conserved amino acids which has structural and functional significance (Chakraborty et al. 2011; Ashkenazy et al. 2010). The presence of the conserved domain not only tells about the functional aspect of the respective protein, but also enables to get an idea of its evolution (Branden and Tooze 1999). It has been well acknowledged that cognate ligand binding and the intracellular N-terminal domain, extracellular C-terminal region are correlated with the conserved amino acid residues (Tanaka et al. 2006). However, very few works on conserved domains of the human death receptors have been reported on the structural aspect (Marchler-Bauer et al. 2005). Networking of member proteins in a family or related to biological pathway of a disease is very much important to understand the drug target discovery (Chakraborty et al. 2010). However, no data are available on the network between human death receptors family.

In this study, we provide information about the conserved domain, amino acid residues and also relate the sequence similarity with the evolutionary divergence of the different death receptors. We also performed molecular phylogenetics to understand the relationship between the family members of death receptors family. We also performed multiple sequences alignment (MSA) to understand sequence similarity and we developed a network to understand associations among the members by using computational biology.

## Materials and methods

### Data collection

We have collected human death receptors gene and protein sequence data as available in the public repository of the National Centre for Biotechnology Information (NCBI) database (Wheeler et al. 2007; Sayers et al. 2011). The protein sequences were collected with the corresponding accession number from the database and analyzed further. The protein sequences collected were in FASTA format for our use.

### Multiple sequences alignment

The protein sequences were analyzed using the well-known multiple alignment tool ClustalW (Chenna et al. 2003) to observe the similarity between the sequences. The graphical output of the tool is visualized using JalView of ClustalW; we have also tried to study using another multiple sequence alignment tool, MUSCLE, to locate the conserved pattern across the sequences of MUSCLE (Edgar 2004). Using the multiple sequence alignment technique, we have observed the similarity in the sequences and their respective alignment scores have been elucidated. In this analysis, seven sequences have been used and TNFR1, Fas, DR3, DR4, DR5, DR6, TNFBR sequences has been represented as Seq1, Seq2, Seq3, Seq4, Seq5, Seq6, Seq7, respectively. We have used notation Seq (*x*:*y*) meaning alignment score between sequence *x*, and sequence *y*.

### Phylogenetic tree and computational analysis

For extensive study of human receptors, we have used POWER (Phylogenetic Web Repeater), a tool based on the concept of ancestral relationship using the genetic distance (Lin et al. 2005). This tool performs multiple sequence analysis and tree building based on ClustalW, PHYLIP (PhyloDraw) (Choi et al. 2000), BLAST and PSI-BLAST (Altschul et al. 1997). A phylogenetic tree (phylogram) is developed to show the distances between protein sequences of human death receptors. We have also developed another phylogenetic tree, i.e., cladogram (ignoring branch length). This cladogram has been used for algorithm analysis based on Aldous as well as Bereg and Wang algorithms (Aldous 1996; Sandvik 2009).

### Conservation pattern of structures and calculation of highly conserved amino acids in human death receptors’ family members

ConSurf Server (Ashkenazy et al. 2010; Glaser et al. 2003) enabled us to calculate the conservation pattern in the structure of human death receptors family (TNFR) members. The conservation scores which have been calculated by ConSurf Server not only discuss the extent of conservation, but also reveal the evolutionary rate. It represents the output in colored format where each conserved position in the chain is represented by different color. It performs the task of sequence to structure relationship by performing multiple sequence analysis, creating phylogenetic tree based on NJ method. It further guides about the rate of evolution of each amino acid residue in the target sequence using either Bayesian method or Maximum likelihood approach.

### Protein–protein network design between the human death receptors

Using STRING (http://string-db.org/), a database of known and predicted protein interactions, we have developed a landscape networking between the human death receptor family members. This web-based database dedicated to protein–protein interactions includes direct (physical) and indirect (functional) associations among the members (Jensen et al. 2009).

## Results

### Collected data

Human death receptor proteins and their genes were compiled using the services provided by the NCBI data bank. Human death receptor genes, their protein IDs, locus, accession number, version, GI have been documented (Table 1).

**Table 1:** Human (Homo sapiens) death receptors and their protein ID have been analyzed in the present study

S. no	Gene symbol	Gene location	Protein Id	Other information	Length
1	TNFRSF1A (TNFR1)	Chromosome: 12; Location: 12p13.2	NP_001056.1	Locus: NP_001056 Definition: tumor necrosis factor receptor superfamily, member 1A precursor [Homo sapiens] Accession: NP_001056 Version: NP_001056.1 GI: 4507575	455 aa
2	FAS (Fas)	Chromosome: 10; Location: 10q24.1	NP_000034.1	Locus: NP_000034 Definition:tumor necrosis factor receptor superfamily member 1B precursor [Homo sapiens] Accession: NP_000034 Version: NP_000034.1 GI: 4507583	335 aa
3	TNFRSF25 (DR3)	Chromosome: 1; Location: 1p36.2	NP_683866.1	Locus: NP_683866 Definition: tumor necrosis factor receptor superfamily member 25 isoform 1 precursor [Homo sapiens] Version: NP_683866.1 GI: 23200021	426 aa
4	TNFRSF10A (DR4)	Chromosome: 8; Location: 8p21	NP_003835.3	Locus: NP_003835 Definition: tumor necrosis factor receptor superfamily member 10A precursor [Homo sapiens] Accession: NP_003835 Version: NP_003835.3 GI: 259906438	468 aa
5	TNFRSF10B (DR5)	Chromosome: 8; Location: 8p22-p21	NP_003833.4	Locus: NP_003833 Definition: tumor necrosis factor receptor superfamily member 10B isoform 1 precursor [Homo sapiens] Accession: NP_003833 Version: NP_003833.4 GI: 224494019	440 aa
6	TNFRSF21 (DR6)	Chromosome: 6; Location: 6p21.1	NP_055267.1	Locus: NP_055267 Definition: tumor necrosis factor receptor superfamily member 10A precursor [Homo sapiens] Accession: NP_003835 Version: NP_003835.3 GI: 259906438	655 aa
7	TNFRSF1B (TNFBR)	Chromosome: 1; Location: 1p36.22	NP_001057.1	Locus: NP_001057 Definition: tumor necrosis factor receptor superfamily member 1B precursor [Homo sapiens] Accession: NP_001057 Version: NP_001057.1 GI: 4507577	461 aa

### Multiple sequence alignment (MSA)

**Fig. 1:**
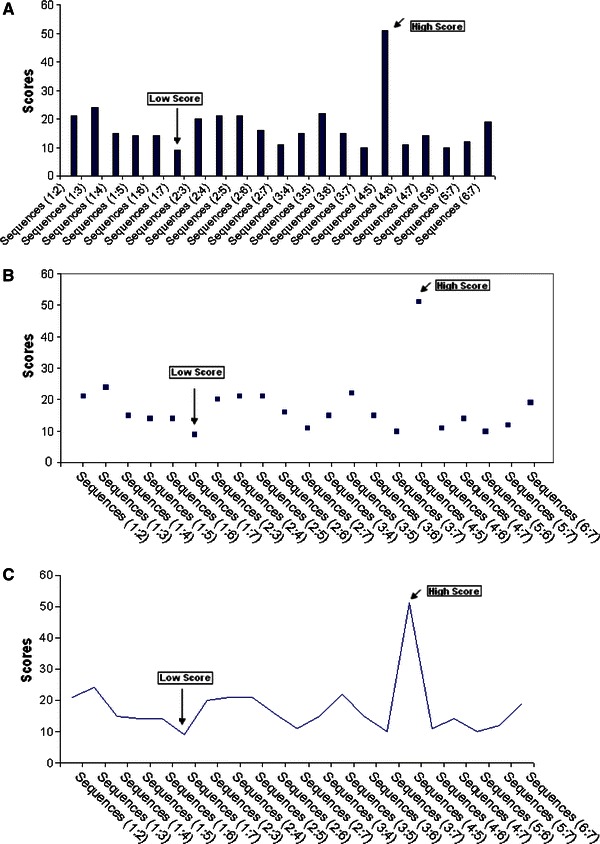
MSA scores of protein sequences of different human death receptors. **a** MSA score between two sequences (the information such as Seq (*x*:*y*) meaning MSA score between sequence *x*, and sequence *y*). **b** Scatter distribution of MSA score, and **c** MSA score connected by *smoothed line* without marker

Multiple sequence alignment was generated to analyze the similarities and differences among the death receptors. The output shows that the sequences share certain conserved regions. These regions were found to be starting from 75–115, 149–151, 190–200, 299–313, 437–451, 493–509, 522–528, 537–549 and 554–566. Certain positions like 181, 233 and 254 were highly conserved. Using multiple sequence alignments, scores have been generated (Fig. 1). The sequence alignment shows highest similarity score of 51 in both the sequences 4 and 5. These results not only indicate the sequence similarity between DR4 and DR5, but also show the excellent sequence match. Lowest similarity score of 9 was observed between the sequences 1 and 7 which illustrate the huge difference between the TNFR1 and TNFBR sequence.

### Phylogenetic tree and computational analysis

**Fig. 2:**
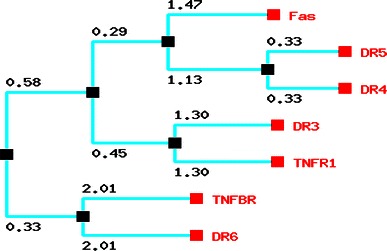
Phylogenetic relationship of the different human death receptors. **a** Using POWER, Phylogenetic Web Repeater, the phylogenetic tree has been constructed

**Fig. 3:**
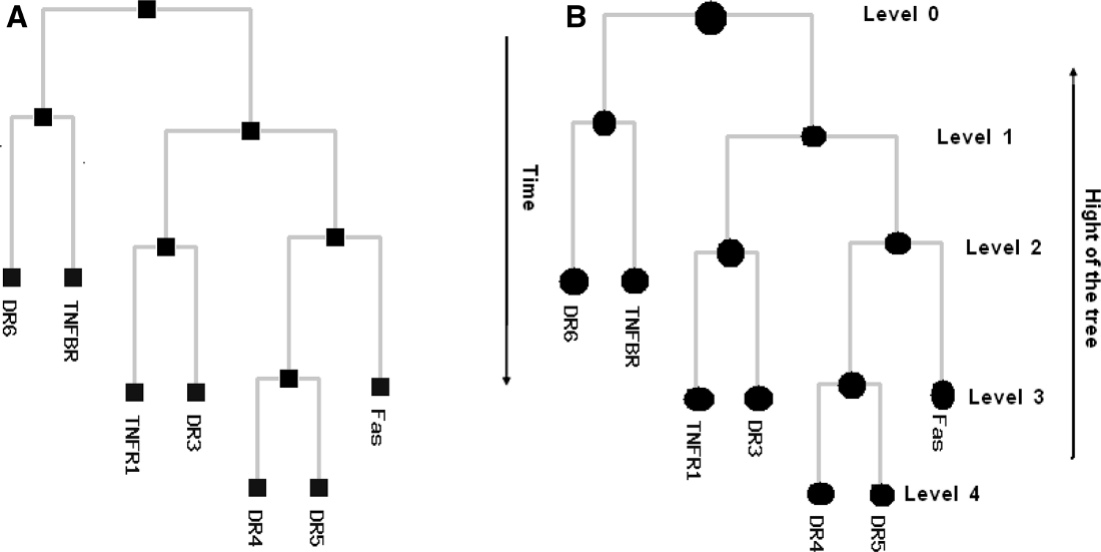
Development of phylogenetic tree. **a** Generated cladogram for tree algorithm analysis. **b** Generated binary tree equivalent to cladogram

The constructed phylogenetic tree is shown in Fig. 2. Phylogenetic tree represents that DR5 and DR4 have same point of origin while on the other hand TNFR1 and DR3 share similar point of origin. The result also depicts that DR6 and TNFBR have same point of origin. The result shows three subgroups according to their common point of origin. Furthermore, we have depicted cladogram (Fig. 3a, b) (“without any distance”) from our phylogram (Fig. 2). From the phylogram, we developed cladogram (Fig. 3a). For the generation of the algorithm, we have depicted a binary tree figure (Fig. 3b) from the cladogram. Here, we assume that the figure is a binary tree and this tree is a level 4 binary tree. The leaf nodes containing DR6 and TNFBR are located at level 2; TNFR1, DR3, Fas at level 3; and DR4, DR5 at level 4, respectively.

### Conservation pattern and calculation of highly conserved amino acids in human death receptors family

**Fig. 4:**
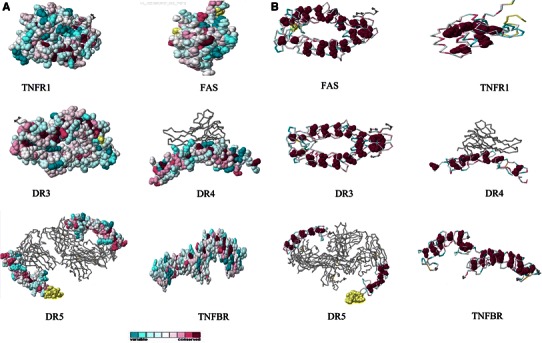
Conservation prototype and backbone structures analysis of the proteins of death receptors. **a** A common conservation prototype with highly conserved amino acids in 3D structure of the death receptors. Amino acid conservation scores have been categorized into nine levels and the color of residue indicates that conservation prototype of the death receptors. **b** Backbone structures of the of the death receptors where we have indicated highly conserved amino acids

**Fig. 5:**
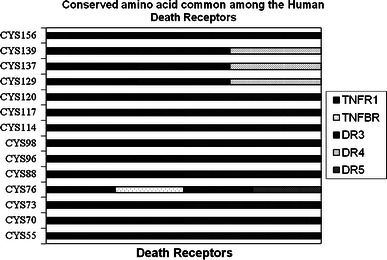
List of amino acid residues which are highly conserved among the death receptors

**Fig. 6:**
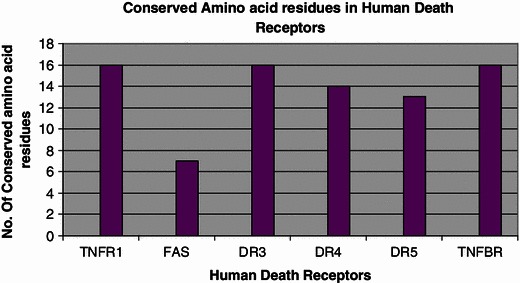
Number of conserved amino acid residues among the death receptors

The structural data comprising the conserved amino acid residues in the human death receptors is represented in the Fig. 4. Here, we have shown conservation patterns in 3D structure and backbone structures with the help of highly conserved residues of death receptors. Figure 5 represents the graphical representation of highly conserved cysteine residues which are common among the death receptors. It describes the position of highly conserved cysteine residues of the amino acid which are common among the different receptors. In this study, the structural data of DR6 has not been predicted by ConSurf Server. Therefore, it could not be included in this paper. We have tried to represent the highly conserved amino acid residues present in each receptor in a separate table (Table 2). We have again recorded the number of highly conserved amino acid residues in death receptors which is represented in Fig. 6. It has been noted from the figure that the highest number of highly conserved residues was shared by TNFR1, DR3 and TNFBR which is 16. On the other hand, the lowest count of 7 was observed in case of Fas for highly conserved residues.

**Table 2:** List of highly conserved residues in human death receptors

S. no	Death receptors	Highly conserved residues
1	TNFR1	CYS43, CYS52, CYS55, CYS70, CYS73, CYS76, CYS88, CYS96, CYS98, CYS114, CYS117, CYS120, CYS129, CYS137, CYS139, CYS156
2	FAS	MET224, ARG234, GLU256, TRP265, LEU278, LEU282, ALA291
3	DR3	CYS43, CYS52, CYS55, CYS70, CYS73, CYS76, CYS88, CYS96, CYS98, CYS114, CYS117, CYS120, CYS129, CYS137, CYS139, CYS156
4	DR4	GLN70, CYS81, GLY84, CYS94, CYS97, CYS113, CYS116, CYS119, CYS129, CYS137, CYS139, CYS153, CYS160, CYS178
5	DR5	CYS28, GLY31, CYS41, CYS44, CYS60, CYS63, CYS66, CYS76, CYS84, CYS86, CYS100, CYS107, CYS125
6	TNFBR	CYS40, GLY43, CYS50, CYS58, CYS61, CYS76, CYS79, CYS83, CYS93, CYS101, CYS103, CYS118, CYS121, CYS127, CYS144, GLY150

### Protein–protein network design between the human death receptors

**Fig. 7:**
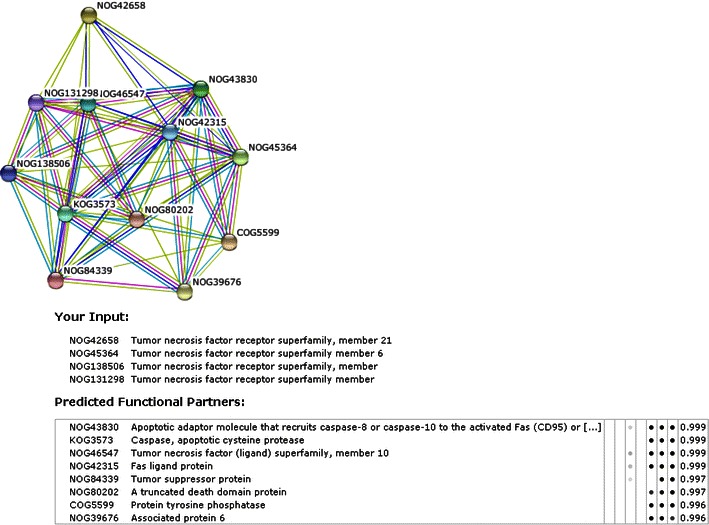
Protein–protein network between the proteins of death receptors. We have used STRING software (http://string-db.org/) for the generation of the network where we provided input as protein of death receptors. It shows a networking layer is not only related between them (protein cascades of the node), but also related to the several other proteins in other signaling pathways

Protein–protein networking was generated between the human death receptors’ family members (Fig. 7). It clearly shows that these proteins are not only interlinked among themselves, but also interlinked with several other proteins. We have also observed from this network that scores of all the nodes ranges from 0.996 to 0.999. Therefore, each node of this network is strongly interconnected.

## Discussion

Apoptosis or programmed cell death is carried out along with several pathways which play a significant role in numerous physiological processes, particularly in development processes. A number of diseases are associated with either excess or inadequate apoptosis, such as AIDS, cancer, and autoimmunity (Krammer 2000; Vaux and Korsmeyer 1999). The two main apoptotic pathways were identified that activate caspases for programmed cell death (Thorburn 2004). One is “intrinsic pathway”, a stress-derived pathway, that involves mitochondrial proteins such as cytochrome *c* (Wang 2001; Huang and Strasser 2000; Cory and Adams 2002) and the other apoptotic pathway is the “extrinsic pathway” that is commenced by stimulus of ‘death receptors’ in the plasma membrane (Hengartner 2000; Sayers 2011). In the second pathway, ligand-bound death receptors, for example, TNF, Fas or TRAIL receptors initiate the process. This pathway was thought to be much easier and well comprehended (Ashkenazi and Dixit 1998). In this case, apoptosis can be started through the stimulation of death receptors which incorporate Fas, TNFRα, DR3, DR4, and DR5 by their respective ligands. Till date, seven known death receptors—Fas, TNFR, DR3, DR4, DR5, DR6, TNFBR—are known to have an intracellular globular protein interaction domain also named as death domain (DD). Ligand binding to the death receptors is perhaps in the form of pre-associated receptor complex (Siegel et al. 2000; Chinnaiyan et al. 1995). The complex activated death receptors hire an adaptor protein entitled Fas-Associated Death Domain (FADD) (Siegel et al. 2000). In this case, we have studied death receptors and their conserved domain, residues as well as evolutionary relations. However, there may be a correlation between conserved domains and Fas-Associated Death Domain (FADD) for death receptors. Chan et al. 2000 established evolutionary relationship between death receptors. Their phylogenetic analysis indicates that the member of the death receptor family represent an ancient divergence. Actually, death domains consisting of 80- 100-residue extended motifs can be seen in cytoplasmic proteins. These proteins belonging to the TNF-receptor super family are trans-membrane proteins and are known by some other names, this has been recorded in (Table 3). Sometime death domains provide as employing modules through their capacity to heterodimerize the DD of distinct proteins, including adaptor proteins such as Fas-associated protein with Death Domain (FADD), TNF Receptor-Associated protein with Death Domain (TRADD) and Receptor Interacting Protein (RIP) (Bridgham et al. 2003). It has been reported that death receptors are characterized by the presence of intracellular death domain (Ryan and Aksentijevich 2009) and this death domain consists of cysteine-rich residues. According to Itoh et al. 1991, members of the TNF-R1 family include 1–5 extracellular cysteine-rich domains. From our study, it is very clear that CYS76 could be considered as one of the highly conserved amino acid which is found to be common among TNFR1, TNFBR, DR3 and DR5, whereas CYS137, CYS129 and CYS139 are only present in TNFR1, DR3 and DR4. CYS76 is the only residue common among death receptors TNFR1, DR3 and DR5. Moreover, we have found that all those residues which are conserved in TNFR1 are also conserved in DR3. Fas seem to be the only death receptor which had none of the conserved residues common to the rest of the human death receptors. Our phylogenetic analysis as well as highly conserved amino acid analysis supports the view of cysteine-rich residues. We have noted that CYS129, CYS137 and CYS139 are shared by the TNFR1, DR3 and DR4. The result of phylogenetic tree as well as alignment scores represents that DR5 and DR4 form one subgroup, while TNFR1 and DR3 forms another subgroup and the third subgroup comprises DR6 and TNFBR. On the other hand, Fas is found to be distant with the rest of the caspase receptors. Phylogenetic analysis validates the point given by the ConSurf Server where the tree is apparently showing the right pathway of diversion as well as evolution because all the amino acid like CYS70, 73, 88, 76, 96, 98, 114, 117, 120, 129, 137, 139, 156 are shown common to both the TNRF1 and DR3. In order to study the conservation pattern in the structure of receptor proteins, we have used ConSurf server. This software enables to explore the 3D structure from the protein sequence data. This server uses the sequence data provided by PDB (Berman et al. 2000) file and further allows the user to go for stepwise calculation of evolutionary conserved residues from closely related homologous amino acid sequences using PSI-BLAST. The rate of evolution is calculated using either distance-based method or character-based method. It also determines the conservation score of a particular amino acid at a particular position. ConSurf server uses either the Maximum likelihood approach or the Empirical Bayesian method (Mayrose et al. 2004) to study the rate of evolution at each position. This tool is quite user-friendly and enables to visualize and analyze the receptor protein structure using its feature First Glance in JMOL.

**Table 3:** Death receptors, their common names and their interaction molecule

S. no.	Death receptors	Synonyms/related protein	Interaction with other molecules	References
1	TNFR1	FPF; p55; p60; TBP1; TNF-R; TNFAR; p55-R; CD120a; TNFR55; TNFR60; TNF-R-I; TNF-R55; MGC19588; TNFRSF1A, DR1	PSMD2, FADD, Tumor necrosis factor-alpha, BAG4, IKK2, Caspase 10, Janus kinase 1, UBE2I, TRPC4AP, PIP4K2B, TRAF2, RIPK1 TRADD	Boldin et al. (1995); Hsu et al. (1996a, b)
2	FAS	APT1; CD95; FAS1; APO-1; FASTM; ALPS1A; TNFRSF6, DR2	FADD, CFLAR, Caspase 10, Caspase 8, Fas ligand, PDCD6	Huang et al. (1996); Thomas et al. (2002); Shu et al. (1997); Jung et al. (2001)
3	DR3	TR3; DDR3; LARD; APO-3; TRAMP; WSL-1; WSL-LR; TNFRSF12; TNFRSF25	TRADD	Kitson et al. (1996)
4	DR4	APO2; CD261; MGC9365; TRAILR1; TRAILR-1; TNFRSF10A	FADD, DAP3	Miyazaki and Reed (2001); Schneider et al. (1997); Marchler-Bauer et al. (2005); Mayrose et al. (2004)
5	DR5	CD262; KILLER; TRICK2; TRICKB; ZTNFR9; TRAILR2; TRICK2A; TRICK2B; TRAIL-R2; KILLER/DR5; TNFRSF10B	FADD, TRAIL, Caspase 10, Caspase 8	Schneider et al. (1997); Chaudhary et al. (1997); Hymowitz et al. (1999)
6	DR6	BM-018; MGC31965; TNFRSF21	N-APP, TRADD	Pan et al. (1998); Nikolaev et al. (2009)
7	TNFBR	p75; TBPII; TNFR2; CD120b; TNFR1B; TNFR80; TNF-R75; p75TNFR; TNF-R-II; TNFRSF1B	TTRAP,TRAF2	Pype et al. (2000); Bouwmeester et al. (2004); Munroe and Bishop (2004); Hostager and Bishop (2002); Carpentier et al. (2008)

## Conclusions

Several fascinating queries about the conserved domains and evolutionary relationship between these receptor proteins need comprehensive understanding. It has been found that all the conserved domains indicate either structural or functional relevance in terms of evolutionary change. So, we performed an in silico study using sequence and structure analysis from the various tools of bioinformatics. Even though the cell death signaling pathways have been studied for the past few years, there is not much data available specifically on the human death receptors, their conserved domains and even with respect to their structures. We know about the pathways and also know a number of the proteins that may be involved in the reaction. But, we have to understand more about the evolutionary relationship as well as structural and functional relationship between these family members. To address this, in silico analysis was carried out to understand the conserved domain, residues, evolutionary relation and landscape networking of death receptors. This work is a preliminary effort to know the structural and functional relationship. In this analysis, we applied a pioneering and quick method to apprehend the structural, functional and phylogenetic association among the death receptors family. However, we have to go long way to understand the structural and functional relationship between the death receptors and further study is required in this area. Current study may provide great help to future researchers to progress on more findings between the structural and functional relationship of the death receptors.

## Abbreviations

TNFR1: Tumor necrosis factor receptor 1
DR: Death receptor
TNFBR: Tumor necrosis factor beta receptor
DD: Death domain
